# Plastid–endomembrane connections in apicomplexan parasites

**DOI:** 10.1371/journal.ppat.1007661

**Published:** 2019-06-13

**Authors:** Michael J. Boucher, Ellen Yeh

**Affiliations:** 1 Department of Microbiology and Immunology, Stanford University School of Medicine, Stanford, California, United States of America; 2 Department of Biochemistry, Stanford University School of Medicine, Stanford, California, United States of America; 3 Department of Pathology, Stanford University School of Medicine, Stanford, California, United States of America; 4 Chan Zuckerberg Biohub, San Francisco, California, United States of America; University of Wisconsin Medical School, UNITED STATES

The apicoplast is a nonphotosynthetic plastid found in most apicomplexan parasites, including *Plasmodium* spp. (causative agents of malaria) and *Toxoplasma gondii* (causative agent of toxoplasmosis) [1, 2]. It houses essential metabolic pathways and is the target of several antiparasitic drugs [3]. A peculiar feature of the apicoplast is that, unlike endosymbiotic organelles in model organisms, it is integrated into the endomembrane system. Here, we discuss the interplay between the apicoplast and molecular machinery typically associated with the endomembrane system, highlighting outstanding questions and opportunities for drug discovery.

## The apicoplast and endomembrane system became uniquely intertwined during secondary endosymbiosis

**Fig 1:**
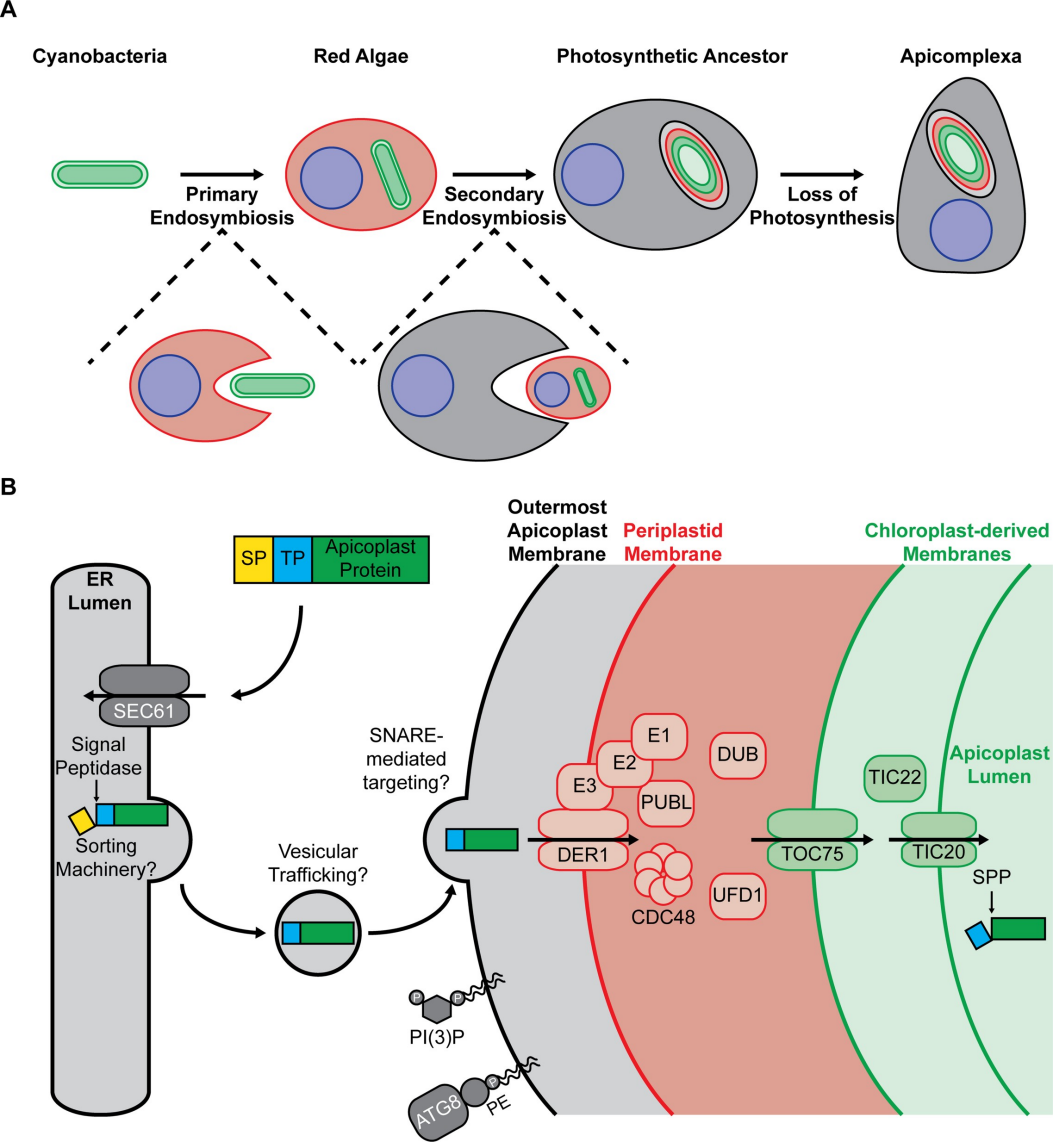
Models for apicoplast evolutionary history and lumenal protein import. (A) Model for apicoplast evolutionary history. Red algae arose following primary endosymbiosis, during which a eukaryotic cell engulfed a photosynthetic cyanobacterium that underwent evolutionary reduction to become a chloroplast. The ancestors of the Apicomplexa emerged following secondary endosymbiosis, during which another eukaryotic cell engulfed a red alga, which then underwent evolutionary reduction to become a four-membraned, photosynthetic secondary plastid. During evolution of the Apicomplexa, the secondary plastid lost its photosynthetic machinery but retained components of key metabolic pathways to become what we now know as the apicoplast. Note that this model is simplified and that the precise evolutionary events that gave rise to the apicoplast (e.g., vertical plastid inheritance from a common chromalveolate ancestor versus acquisition by tertiary endosymbiosis) are not yet resolved. (B) Model for import of lumenal apicoplast proteins via the secretory system and retooled ERAD machinery. Most apicoplast proteins contain a bipartite N-terminal targeting signal consisting of a eukaryotic SP followed by a plant-like TP. The SP mediates cotranslational import into the ER via the SEC61 complex and is cleaved by the signal peptidase complex to reveal the TP. The TP then mediates sorting and trafficking to the apicoplast and import across its membranes. The machinery involved in recognizing apicoplast proteins in the endomembrane system is unknown. Apicoplast proteins are presumed to traffic from the ER to the outermost apicoplast membrane via a vesicular trafficking pathway. After crossing the outermost apicoplast membrane, apicoplast proteins cross the periplastid membrane using retooled ERAD machinery. Finally, lumenal apicoplast cargo crosses the innermost apicoplast membranes via complexes related to the TOC and TIC machinery of primary plastids. The apicoplast outer membrane contains PI(3)P and ATG8, which are associated with the endomembrane system in model systems. ATG8, autophagy-related 8; CDC48, cell division cycle 48; DER1, degradation in the ER 1; DUB, deubiquitinase; ER, endoplasmic reticulum; ERAD, ER-associated degradation; P, phosphate; PE, phosphatidylethanolamine; PI(3)P, phosphatidylinositol 3-phosphate; PUBL, plastid ubiquitin-like protein; SNARE, soluble N-ethylmaleimide–sensitive factor attachment protein receptor; SP, signal peptide; SPP, stromal processing peptidase; TIC, translocon of the inner chloroplast membrane; TOC, translocon of the outer chloroplast membrane; TP, transit peptide; UFD1, ubiquitin fusion protein degradation 1.

Apicomplexan parasites evolved from photosynthetic algae that acquired a plastid through successive endosymbiotic events. First, primary endosymbiosis gave rise to chloroplasts (primary plastids) when a eukaryotic cell engulfed a cyanobacterium that over time became a permanent fixture of the photosynthetic cell (Fig 1A). Then, a chloroplast-containing red alga was itself engulfed by another eukaryote to establish a complex secondary plastid during secondary endosymbiosis. One lineage containing a red algal secondary plastid adopted a parasitic lifestyle and gave rise to the Apicomplexa, and the apicomplexan plastid, or apicoplast, was retained in these pathogens despite loss of photosynthesis. Notably, the precise origins of the apicoplast and other red algal secondary plastids are unclear, with significant debate as to whether all such plastids originated from a single endosymbiotic event and have been acquired vertically ever since (the chromalveolate hypothesis) [4] or whether some lineages acquired plastids through more complex processes such as tertiary endosymbiosis [5]. For in-depth discussions of models for plastid evolution, we direct the reader to reviews on the subject [6–10].

One striking result of secondary endosymbiosis is that the apicoplast is bound by four membranes (Fig 1B). Based on the current working model for secondary endosymbiosis (Fig 1A) and the fact that apicoplast protein import involves machinery homologous to the translocons of the outer and inner chloroplast membranes (TOC and TIC complexes) of primary plastids [11–13], the inner two apicoplast membranes are thought to be of cyanobacterial origin. The origins of the two outer membranes, however, are unclear. The most commonly described model proposes that the second plastid membrane from the outside, called the periplastid membrane, is derived from the red algal plasma membrane, and the outermost plastid membrane is derived from endomembrane of the host cell [14]. However, other models, such as an endomembrane origin for both membranes, have been proposed and are equally plausible [10]. Irrespective of the precise origins of the outer plastid membranes, secondary endosymbiosis inextricably linked red alga–derived plastids to the host endomembrane system. In fact, the secondary plastid of chromists actually resides within the endoplasmic reticulum (ER), with the outermost plastid membrane contiguous with the ER membrane [15, 16]. This contiguity does not exist in the Apicomplexa, in which the ER and apicoplast are discrete organelles [17, 18]. However, the apicoplast and ER membranes have been visualized in close apposition to one another, indicating that there may be physical contact between these compartments [17, 19].

### Apicoplast protein import occurs via the ER

Transfer of endosymbiont genes to the host nucleus is a hallmark of endosymbiosis and necessitates a pathway by which nuclear-encoded proteins are imported back to the organelle. In the Apicomplexa, nuclear-encoded apicoplast proteins traffic via the secretory system [20]. Most apicoplast proteins utilize a bipartite N-terminal targeting sequence consisting of a canonical eukaryotic signal peptide (SP) followed by a chloroplast-derived transit peptide (TP) [20, 21]. The SP mediates import into the ER, after which the TP mediates sorting and trafficking to the apicoplast (Fig 1B). TPs are best studied in *Plasmodium falciparum* and are highly diverse, sharing only the general features of being enriched in basic amino acids and depleted in acidic amino acids, having putative heat shock protein 70 (HSP70) binding sites, and being unstructured in vitro [22, 23].

The mechanism by which degenerate TPs enable specific sorting and trafficking to the apicoplast is poorly understood. Disruption of ER-to-Golgi protein trafficking with the fungal toxin brefeldin A does not ablate import of lumenal apicoplast proteins [24, 25], suggesting a model wherein recognition and sorting occur pre-Golgi in the ER. However, other data support a potential role for the Golgi in trafficking lumenal apicoplast proteins [26], indicating that there is still much to learn about this sorting process. In particular, identification of candidate machinery that carries out recognition and sorting of apicoplast-bound cargo in the ER will be critical for elucidating this pathway.

After TP recognition, it is additionally unknown how proteins traffic from the endomembrane system to the outermost apicoplast membrane. Although a vesicular trafficking route seems most likely, other models, such as direct transfer of proteins via organelle–organelle contact sites, have not been ruled out. Disruption of SNARE disassembly by expression of a dominant negative α-SNAP phosphomutant causes apicoplast vesiculation in *T*. *gondii* [27], consistent with a vesicular model involving SNARE-mediated delivery of cargo. Additionally, in *P*. *falciparum* parasites induced to lose their apicoplasts, lumenal apicoplast proteins localize to diffuse puncta that may represent stalled vesicle-trafficking intermediates [28], although the transport competence of these vesicles has not been shown. Similarly, vesicles containing apicoplast outer-membrane proteins have been observed in *T*. *gondii* under both apicoplast-intact and -disrupted conditions [29–33]. Because apicoplast outer-membrane proteins tend to lack TPs [34] and lumenal apicoplast proteins are absent from these outer-membrane protein–containing vesicles [33], these vesicles suggest two distinct trafficking pathways: one TP-dependent for lumenal proteins and one TP-independent for outer-membrane proteins. Overall, current evidence for vesicle-mediated trafficking of apicoplast cargo is circumstantial, and there is therefore significant need for detailed characterization of the trafficking routes and molecular machinery involved in this process.

### Import across the periplastid membrane involves borrowed ER machinery

Once a lumenal apicoplast protein is delivered to the outermost apicoplast membrane, the next step is to cross the periplastid membrane. To accomplish this, the Apicomplexa have retooled the ER-associated degradation (ERAD) pathway, which is a conserved eukaryotic pathway typically used for retrotranslocating misfolded proteins from the ER to the cytoplasm for degradation by the ubiquitin–proteasome system. Apicomplexans not only retain canonical, ER-localized ERAD machinery but also encode a nearly complete, divergent set of apicoplast-localized proteins, including DER1-like proteins (potentially constituting the translocon), the AAA ATPase CDC48 (thought to provide the mechanical power for protein translocation), a plastid ubiquitin-like protein (PUBL), and E1/E2/E3 ubiquitin ligases, among others (Fig 1B) [35–41]. DER1 and CDC48 are essential for apicoplast protein import in *T*. *gondii* [38, 41], confirming the importance of this borrowed ER machinery for apicoplast biology. Interestingly, PUBL and the E2 ubiquitin-conjugating enzyme are also essential for apicoplast protein import in *T*. *gondii* [40, 41], but whether ubiquitylation of apicoplast cargo actually occurs in cells is unclear. In canonical ERAD, ubiquitylation tags retrotranslocated proteins for degradation, so it is unknown what function ubiquitylation would serve during apicoplast protein import. After crossing the periplastid membrane, apicoplast protein import transitions to utilizing canonical chloroplast pathways, with translocation across the inner two apicoplast membranes involving the TOC and TIC complexes of primary plastids [11–13].

### Autophagy machinery and phosphoinositides may have roles in apicoplast segregation

Another intriguing apicoplast–endomembrane connection is the localization of the autophagy protein ATG8 and phosphoinositides (PIs) to the outer apicoplast membrane (Fig 1B) [32, 42–46]. Here, these important membrane markers may have roles in apicoplast biogenesis, the process whereby new apicoplasts are replicated from an existing apicoplast and are segregated into new daughter cells during parasite replication. In model systems, ATG8 family proteins are localized to autophagosomes, which are specialized organelles that degrade cellular constituents and are thought to derive from multiple endomembranes [47]. Apicoplast-localized ATG8 is essential in both *T*. *gondii* and *P*. *falciparum* [48, 49]. Specifically, *T*. *gondii* ATG8 (*Tg*ATG8) appears to mediate association of the newly replicated apicoplasts with centrosomes, facilitating their segregation into daughter parasites [48]. Knockdown of *P*. *falciparum* ATG8 (*Pf*ATG8) in malaria parasites also supports a function in apicoplast fission and/or segregation [49]. In fact, in blood-stage *P*. *falciparum*, the only essential role of *Pf*ATG8 is its apicoplast function [49], while it is still debated whether canonical macroautophagy occurs at all in this stage [50]. The precise mechanisms by which ATG8-mediated centrosome association promotes faithful apicoplast segregation and whether ATG8 has essential nonapicoplast function(s) in *T*. *gondii* or in *P*. *falciparum* sexual, mosquito, or liver stages remain unknown.

Phosphatidylinositol is a lipid synthesized in the ER and phosphorylated into various PIs that are critical for membrane signaling and dynamics in eukaryotic cells [51]. Similar to ATG8, PIs in the apicoplast membranes may have a role in apicoplast segregation. Depletion of *T*. *gondii* phosphoinositide 3-kinase (*Tg*PI3K) or phosphoinositide kinase, FYVE-type zinc finger containing (*Tg*PIKfyve), which respectively produce phosphatidylinositol 3-phosphate (PI[3]P) and phosphatidylinositol 3,5-bisphosphate (PI[3,5]P_2_), causes apicoplast enlargement followed by apicoplast loss [52]. These data, combined with the apparent absence of a specific protein import defect following *Tg*PI3K or *Tg*PIKfyve depletion [52], are consistent with a role for PIs in a late step of apicoplast biogenesis.

In fact, it is possible that the apicoplast biogenesis functions of ATG8 and PIs are linked, as the autophagy-related protein ATG18 from both *T*. *gondii* and *P*. *falciparum* binds PIs [53, 54]. The human ATG18 homologs WIPI1 and WIPI2 are required for conjugation of the mammalian ATG8 homolog, LC3, to phosphatidylethanolamine (PE) [55]. Consistent with a conserved function, ATG18 depletion in either *T*. *gondii* or *P*. *falciparum* reduced ATG8 lipidation and membrane localization, resulting in an apicoplast biogenesis defect [53]. This defect could not be complemented with a mutant deficient in PI binding [53], specifically linking ATG18 PI binding to its apicoplast biogenesis function. Thus, the current data implicate both autophagy machinery and PIs in a critical step of apicoplast biogenesis, whereas further investigation will uncover their exact molecular mechanisms.

### Apicoplast–endomembrane connections may yield novel antiparasitic drug targets

In addition to its fascinating biology, the retooling of endomembrane machinery during secondary endosymbiosis may provide valuable antiparasitic targets. For example, specific small-molecule inhibitors have been developed against the human homologs of CDC48 and the E1 ubiquitin-activating enzyme as anticancer targets [56–60], providing proof of principle of their utility as potential targets. Furthermore, ATG7 is an essential protein that is required for apicoplast biogenesis [61, 62], presumably via its canonical role as an E1 ligase for ATG8 activation, and may be druggable because of its shared chemistry with E1 ubiquitin-activating enzymes. Finally, inhibitors that disrupt the protein–protein interaction between *Pf*ATG8 and its E2 ligase, *Pf*ATG3, have also been under investigation and may represent a viable antiparasitic strategy [63–65].

In addition to these pathways for which mammalian homologs are established drug targets, we expect that deeper exploration of the interplay between the apicoplast and the endomembrane system will yield additional candidates. For example, the as-yet undiscovered machinery for recognition, sorting, and trafficking of apicoplast cargo may be druggable, as could other currently unidentified biogenesis factors that arose during integration of the apicoplast into the endomembrane system. Therefore, we expect that continued dissection of apicoplast biogenesis mechanisms will elucidate important evolutionary cell biology and will help to sustain a pipeline of novel antiparasitic targets.
